# RPiRLS: Quantitative Predictions of RNA Interacting with Any Protein of Known Sequence

**DOI:** 10.3390/molecules23030540

**Published:** 2018-02-28

**Authors:** Wen-Jun Shen, Wenjuan Cui, Danze Chen, Jieming Zhang, Jianzhen Xu

**Affiliations:** 1 Department of Bioinformatics, Shantou University Medical College, Shantou 515000, Guangdong, China; wjshen@stu.edu.cn (W.-J.S.); d_z_chen@stu.edu.cn (D.C.); 16jmzhang@stu.edu.cn (J.Z.); 2 Computer Network Information Center, Chinese Academy of Sciences, Beijing 100190, China; wenjuancui@cnic.cn

**Keywords:** Protein-RNA interactions, lncRNA-protein interaction networks, derived kernel, regularized least squares

## Abstract

RNA-protein interactions (RPIs) have critical roles in numerous fundamental biological processes, such as post-transcriptional gene regulation, viral assembly, cellular defence and protein synthesis. As the number of available RNA-protein binding experimental data has increased rapidly due to high-throughput sequencing methods, it is now possible to measure and understand RNA-protein interactions by computational methods. In this study, we integrate a sequence-based derived kernel with regularized least squares to perform prediction. The derived kernel exploits the contextual information around an amino acid or a nucleic acid as well as the repetitive conserved motif information. We propose a novel machine learning method, called RPiRLS to predict the interaction between any RNA and protein of known sequences. For the RPiRLS classifier, each protein sequence comprises up to 20 diverse amino acids but for the RPiRLS-7G classifier, each protein sequence is represented by using 7-letter reduced alphabets based on their physiochemical properties. We evaluated both methods on a number of benchmark data sets and compared their performances with two newly developed and state-of-the-art methods, RPI-Pred and IPMiner. On the non-redundant benchmark test sets extracted from the PRIDB, the RPiRLS method outperformed RPI-Pred and IPMiner in terms of accuracy, specificity and sensitivity. Further, RPiRLS achieved an accuracy of 92% on the prediction of lncRNA-protein interactions. The proposed method can also be extended to construct RNA-protein interaction networks. The RPiRLS web server is freely available at http://bmc.med.stu.edu.cn/RPiRLS.

## 1. Introduction

The interactions of proteins with other proteins, peptides, DNAs and RNAs govern most the essential molecular function. RNA-protein interactions (RPIs) have a critical influence on post-transcriptional gene regulation [1,2,3], viral assembly [4,5,6], cellular defence [7], protein synthesis [8,9] and various other fundamental biological processes [10,11]. A significant portion of transcripts is long non-coding RNAs (lncRNAs) which are not translated into proteins and are longer than 200 nucleotides [12]. LncRNAs normally function with their interacting proteins [13]. For instance, the lncRNA HOTAIR regulated the HOXD locus in *trans* by interacting with PcG proteins [14]; several lncRNAs were shown to be able to interact with AUF1, a protein linked to aging and cancer [15]; lncRNAs binding to JARID2 protein were essential for the recruitment of PRC2 to the chromatin [16]; lncRNA GAS5 inhibited hepatitis C virus replication by decoying HCV NS3 protein [17]. Hence, the study of RPIs is essential for understanding their functions. Compared to those of protein-protein interactions and DNA-protein interactions, current knowledge regarding RNA-protein interactions, especially lncRNA-protein interactions is still limited. In this study, we propose a novel machine learning method, which we call RNA-protein interaction prediction based on regularized least squares (RPiRLS), to quantitatively predict the potential RNA-protein interactions.

The experimental determination of RPIs remains expensive and time-consuming [18,19,20], but fortunately, the accumulated RPI experimental data facilitate the development of computational models for RPI prediction [21,22,23]. In 2011, Pancaldi and Bähler [24] introduced a computational approach for RBP (RNA binding protein)-mRNA interaction prediction. They employed Support Vector Machines (SVMs) and Random Forests (RFs) based on more than 100 physical and functional features of RPIs, including gene ontology, chromosomal position, gene and protein physical properties, protein localization, experimental translation, mRNA properties, predicted protein structure, UTR properties and genetic interactions. Bellucci et al. [25] proposed a method called catRAPID for the prediction of protein lncRNA interaction. They evaluated the interaction propensities of protein-RNA based on their physicochemical properties, including secondary structure, hydrogen bonding and van der Waals. Muppirala et al. [26] developed a method called RPISeq, which predicted RPIs solely based on primary sequences. The RPISeq method still employed SVMs and RFs but exploited different features. They represented each sequence of proteins and RNAs as the normalized frequencies of the corresponding 3-mer and 4-mer, respectively. In 2013, based on the same feature vectors presented in Muppirala et al., Wang et al. [27] first reduced the dimensionality of feature vectors, and then performed the RPIs prediction by using naive Bayes classifier which assumed the independence of attributes and by using extended naive Bayes classifier which considered the correlation between attributes. Lu et al. [28] integrated the information on the secondary structure, hydrogen bonding propensities and Van der Waals of lncRNAs and proteins with Fisher’s linear discriminant model. In 2015, Suresh et al. [29] developed a method called RPI-Pred to predict RPIs by considering the high-order 3D protein and RNA structure information. In 2016, Pan et al. [30] proposed a new method named IPMiner that integrated deep learning with stacked ensembling to improve the prediction performance of ncRNA-protein interactions.

In this paper, we classified RNA-protein pairs as interacting or non-interacting by integrating derived kernel with regularized least squares (RLS) [31]. The motivation is to relate the sequence information of proteins and RNAs to their biological functions, i.e., interactions. Our method attempted to extract discriminant subsequence features from amino acid sequences and nucleotide sequences. The derived kernel measures the similarity between two biological sequences by capturing nucleic acid or amino acid compositions and repetitive sequence patterns. We used regularized least squares in learning as the computations performed by RLS algorithms can be expressed using just inner products, hence allowing efficient implementation of kernel-based learning, in addition the RLS algorithms often perform comparable to the best batch classifiers [32]. Since the dimensionality of feature space increases exponentially with the template size, for computational sake, we set upper limit for template size. On the other hand, we categorized 20 amino acids into several groups based on their physiochemical properties [33,34,35], the reduced alphabet representation of the protein sequence allows larger template size and also decreases the dimensionality of feature space.

## 2. Results

### 2.1. Parameter Selection for RLS

We considered the derived kernel with two-layer architecture, hence there were two template sets, denoted as $T^{P}$ and $T^{R}$ needed to be constructed for protein and RNA, respectively. Here we considered all possible substrings of the same length making up a template set. The template set $T^{P}$ for amino acid sequences was composed of substrings with *k* continuous amino acids, while the template set $T^{R}$ for nucleic acid sequences was composed of substrings with *l* continuous nucleic acids. In order to extract discriminant subsequence features from amino acid sequences and nucleotide sequences, we explored the effect on RPI prediction over a range of choices for the template sizes of protein and RNA. The training set RPI2662 was used to determine these parameters. In our case, we used different template sizes of protein and RNA chosen from set {1,2,…,4}∪{1,2,…,8} for RPiRLS and {1,2,…,6}∪{1,2,…,8} for RPiRLS-7G. With different combination of protein template size and RNA template size, the combined kernel ${\hat{K}}_{2}^{\mathrm{dk}}$ was integrated with RLS to predict RNA-protein interactions. The ten-fold stratified cross-validation has been verified to be the best algorithm for model selection on a large scale experiments [36], therefore on the data set RPI2662, we tuned the parameter λ by ten-fold stratified cross-validation with the optional parameter set $\left\{\lambda =e^{n},\;n=-15,\;\cdots ,15\right\}$. The data set RPI2662 was divided into ten mutually exclusive folds and the mean response of each fold was approximately equal. In each test we merged 9 parts of the samples as the training set and left the other part as the test set. The parameter λ was chosen by leave-one-out cross-validation on the training set. For RPiRLS, in all the ten sets, $\lambda =e^{-2}$ uniformly achieved the best performance in the training data. Table 1 and Table 2 showed the performance of the proposed method in terms of AUC and accuracy with different combination of parameters *k* and *l*, respectively. The experiment results showed that when the protein template size k=2 and the RNA template size l=5, the model performs best with AUC score of 0.926 and accuracy of 0.830. The other measurements of specificity (SP) and sensitivity (SE) were 0.771 and 0.890, respectively. While for RPiRLS-7G, $\lambda =e^{-3}$ uniformly performed best in all the ten sets. Table 3 showed that the method achieved the best prediction accuracy of 0.823 when the protein template size k=3 and the RNA template size l=4. The other measurements (AUC, SP and SE) were observed as 0.902, 0.761 and 0.884, respectively. The computational results showed that the RPiRLS classifier outperformed the RPiRLS-7G classifier in terms of various performance measurements, indicating that the diversity of amino acids at a sequence is important for the prediction of RPIs.

### 2.2. Performance on Predicting RNA-Protein Interactions with Known Structures

In order to evaluate the reliability and robustness of RPiRLS and RPiRLS-7G, we compared them with other two state-of-the-art methods RPI-Pred and IPMiner. The RPI2241 and RPI369 data sets after removing overlapping RPIs with the training data were evaluated. Both the RPiRLS and RPiRLS-7G classifiers were trained on the RPI2662 data set, and tested on the RPI2241 and RPI369 data sets, respectively.

As shown in Table 4 and Table 5, RPiRLS outperformed the RPiRLS-7G, RPI-Pred and IPMiner methods on both data sets. For the RPI369 data set as shown in Table 4, the performance of the RPiRLS method was 0.85, 0.92, 0.84 and 0.86 for predictive accuracy, AUC, specificity and sensitivity, respectively. While the predictive accuracy of the RPI-Pred and IPMiner methods were just 0.49 and 0.5, respectively which were much lower than RPiRLS’s. The remaining measurements (specificity and sensitivity) were observed as 0.34 and 0.63, respectively for RPiRLS, and 0.52 and 0.48, respectively for IPMiner. The RPiRLS method outperformed RPI-Pred and IPMiner in terms of accuracy, specificity and sensitivity on the RPI369 data set.

Similar results were observed on the RPI2241 data set in Table 5. The specificity of the RPI-Pred and IPMiner methods was just 0.38 and 0.20, respectively, indicating there was a positive bias in their predictions of performance. A low specificity increases the labor, cost, and time needed to perform the required experimental tests, but our RPiRLS method achieved both reasonable specificity and sensitivity.

Furthermore, we evaluated the RPiRLS classifier on large-scale RNA-protein pairs in the currently available RPIntDB data base. The RPiRLS method correctly predicted 35980 out of 43010 RPIs, reaching the predictive accuracy of 84%.

### 2.3. Performance on Predicting ncRNA-Protein Interactions

To explore the effectiveness of the proposed method on predicting ncRNA-protein interactions, a large-scale ncRNA-protein interaction data set (we called NRPI13153) was retrieved from the NPInter data base [37]. We trained RPiRLS and RPiRLS-7G on the RPI2662 data set, and tested it on the NRPI13153. Table 6 showed the prediction results compared with the RPI-Pred classifier on the NRPI13153 data set. The IPMiner method showed a significantly positive bias on predicting ncRNA-protein interactions, thus here we didn’t include it into the comparison. The predictive accuracy for different organisms were separately computed. For the six organisms, our method RPiRLS performed best for the *Homo sapiens* and *Saccharomyces cerevisiae*, RPI-Pred performed best for *Drosophila melanogaster*, *Escherichia coli* and *Mus musculus*, and both methods obtained the same predictive accuracy for the *Caenorhabditis elegans*. RPiRLS outperformed RPiRLS-7G over all six organisms. For 13153 ncRNA-protein pairs, the RPiRLS method achieved an accuracy of 91% compared to 76% for RPiRLS-7G and 88% for the RPI-Pred method. We further tested RPiRLS and RPiRLS-7G on the LNRPI12114 data set which was a subset of the NRPI13153 data set and consisted of only lncRNA-protein interactions (lncRPIs). Our model achieved an overall accuracy of 92% compared to 77% for RPiRLS-7G and 89% for the RPI-Pred classifier as shown in Table 7. The predictive performance of RPiRLS was improved in 5 out 6 organisms compared with its performance on the NRPI13153 data set. The results indicated the effectiveness of our method to predict lncRNA-protein interactions only by using primary sequences of proteins and RNAs.

### Performance on Predicting LncRNA-Protein Interaction Networks

Predicting lncRNA-protein interaction networks is useful to explore the molecular mechanisms that are regulated by lncRNAs [38,39]. In this experiment, we evaluated the performance of RPiRLS on building lncRPI networks and further compared its performance with RPI-Pred. On the basis of the data in NPInter, we analyzed the results of four organisms, i.e., *Caenorhabditis elegans*, *Drosophila melanogaster*, *Escherichia coli* and *Saccharomyces cerevisiae*, consisting of 4, 61, 78 and 437 lncRPIs, respectively.

**Figure 1:**
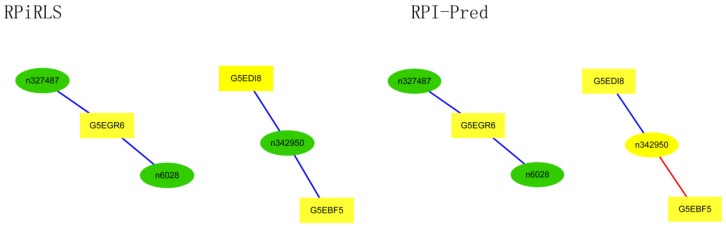
Comparison of the long non-coding RNA-protein interaction networks predicted by the RPiRLS and RPI-Pred methods, for *Caenorhabditis elegans.* Networks are visualized with Cytoscape v3.4.0. The green (ellipse) and yellow (rectangle) nodes representing lncRNAs and proteins respectively, are connected by edges (solid lines) indicating an interaction between them. The edges colored in blue and red indicate true positive and false negative interactions, respectively.

**Figure 2:**
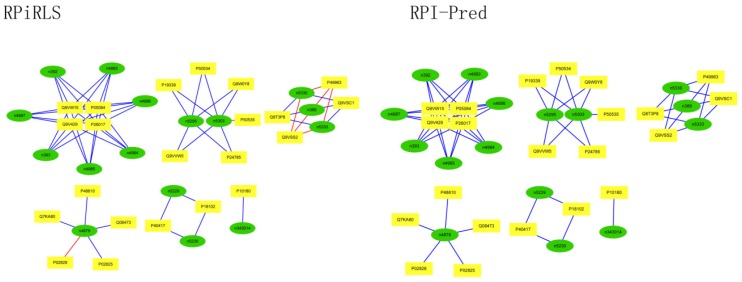
Comparison of the long non-coding RNA-protein interaction networks predicted by the RPiRLS and RPI-Pred methods, for *Drosophila melanogaster*.

**Figure 3:**
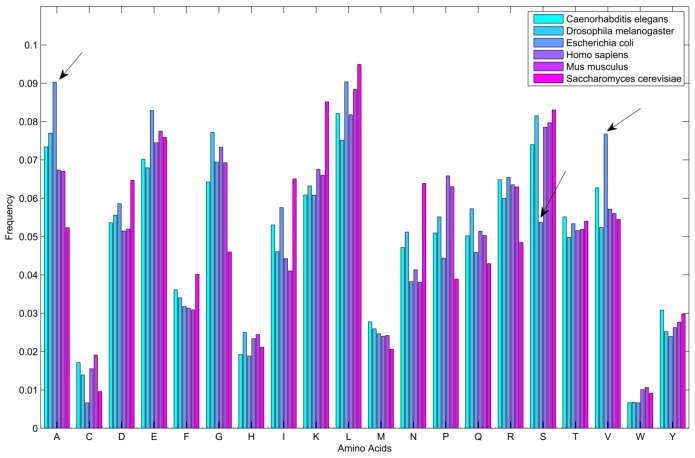
Histogram of the amino acid observing frequencies of lncRNA-binding proteins for six organisms. *Escherichia coli* has relative higher observing frequencies of amino acids A and V as well as much lower content of amino acid S compared with that of the other five organisms (highlighted by arrows).

**Figure 4:**
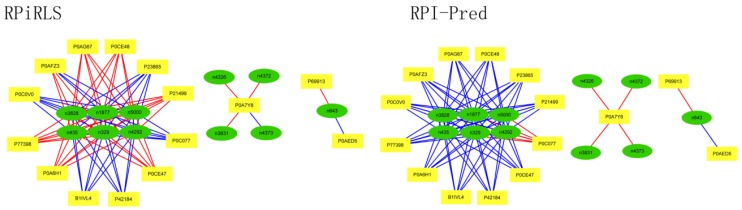
Comparison of the long non-coding RNA-protein interaction networks predicted by the RPiRLS and RPI-Pred methods, for *Escherichia coli*.

**Figure 5:**
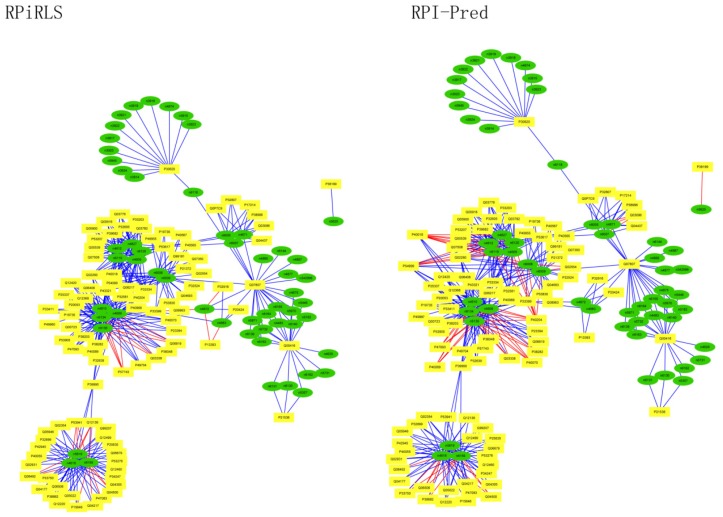
Comparison of the long non-coding RNA-protein interaction networks predicted by the RPiRLS and RPI-Pred methods, for *Saccharomyces cerevisiae*.

For *Caenorhabditis elegans*, the RPiRLS method correctly identified all 4 lncRPIs (blue edges) while the RPI-Pred method correctly identified 3 out of 4. As shown in Figure 1, RPI-Pred made incorrect prediction for the pair of n342950-G5EBF5 (red edges). In Figure 2, RPI-Pred correctly predicted all 61 lncRPIs, whereas RPiRLS missed 7 out of 61 lncRPIs for *Drosophila melanogaster*. These 6 out of 7 incorrect predictions which were observed between two proteins P49963 and Q9VSS2 (yellow rectangle) and three signal recognition particle (SRP) RNAs n5330, n5333 and n389 (green ellipse), formed the SRP RNA-protein interactions. For *Escherichia coli*, the RPiRLS classifier made much more errors than the RPI-Pred method, with predictive accuracies of 45% vs. 86%. The performance of RPiRLS for *Escherichia coli* was much poorer than that for the other five organisms. In order to analyze why RPiRLS had relative poor performance on *Escherichia coli*, we estimated the amino acid composition of *Escherichia coli* compared with that of the other five organisms. As shown in Figure 3, we found that *Escherichia coli* had relative higher observing frequencies of Alanine and Valine as well as much lower content of Serine compared with that of the other five organisms. The amino acid composition bias in *Escherichia coli* probably leaded to poor results. As shown in Figure 4, among 43 incorrect predicted pairs, 39 RPIs corresponded to 7 protein hubs, e.g., P0A6H1, P0AFZ3, P0AG67, P0CE47, P0CE48, P21499 and P77398, each of which appearing as a yellow rectangle node was shown to interact with six transfer-messenger RNAs (tmRNAs), e.g., n3828, n1877, n5000, n435, n329 and N4292 (green ellipse). For *Saccharomyces cerevisiae*, as showed in Figure 5, among 27 incorrect predicted pairs, 10 RPIs were involved in 3 protein hubs (P57743, Q03338 and Q06819), in which each protein interacted with 4 small nuclear RNAs (snRNAs: n4610, n6134, n4606 and n6136), and other 13 RPIs corresponded to 7 protein hubs (P15646, P47083, P53941, Q04217, Q04500, Q08492 and Q12136), each of which interacted with three small nucleolar RNAs (snoRNAs: n5819, n4618 and n6159). The RPiRLS classifier correctly identified 410 out of 437 RPIs, achieving a high accuracy of 94%, compared of 87% (correctly predicted 379 out of 437 pairs) for RPI-pred. In this work, we illustrated the effectiveness and reliability of RPiRLS in predicting RPIs for eukaryotic organisms in networks which comprised a variety protein hubs and RNA hubs.

## 3. Discussion

Mammalian cells contain more than 1000 different proteins interacting with RNA [3]. Normally, any individual RNA can interact with multiple proteins [11,40]. Conversely, most proteins are capable of interacting with multiple RNAs [41]. Given the number of RNAs and RNA-binding proteins, the number of possible RPIs is enormous. High-throughput sequencing methods have accumulated huge amount of RNA-protein binding experimental data and opened new possibilities to measure and understand RNA-protein interactions by computational methods. Most of the previous computational works on RPIs focus on the prediction of RNA-binding proteins or RNA-binding residues in a protein sequence [34,42,43,44]. To our knowledge, very limited works have been developed to predict the specific associations between RNAs and proteins, which play a critical role in post-transcriptional gene regulatory networks. Complex networks of RPIs mediate post-transcriptional gene regulation and therefore prediction of RPIs helps us to gain insight into regulatory networks [45,46].

The work presented here provided a computational method, called RPiRLS, to classify RNA-protein pairs as interacting or non-interacting by integrating a sequence-based derived kernel with regularized least squares. The derived kernel exploited the contextual information around an amino acid or a nucleic acid as well as the repetitive conserved motif information. Our results demonstrated that only the sequence structures of RNAs and proteins provide sufficient information to accurately predict RNA-protein interactions, especially long non-coding RNA-protein interactions. Specifically, the RPiRLS classifier considered each protein sequence comprising up to 20 diverse amino acids, while the RPiRLS-7G classifier encoded each protein sequence by using the 7-letter reduced alphabets according to amino acid physiochemical properties. The computational results showed that the RPiRLS classifier was superior to the RPiRLS-7G classifier in reliability and effectiveness, indicating that the diversity of amino acids at a sequence has critical impact on the function of RNA-binding proteins. On two non-redundant benchmark data sets extracted from the PRIDB, the RPiRLS method outperformed RPI-Pred and IPMiner in terms of accuracy, specificity and sensitivity. Compared with RPI-Pred and IPMiner, the RPiRLS method obtained a reasonable sensitivity at a lower false positive rate. Further, RPiRLS achieved an accuracy of 92% compared to 89% for RPI-Pred on the prediction of lncRNA-protein interactions. The RPiRLS method can be extended to construct RNA-protein interaction networks and therefore helps us to gain insights into post-transcriptional gene regulation.

The reason for the good performance of the proposed method may be due to several factors. Firstly, the use of similarity scores is a significant conceptual change in protein/RNA evaluation, quantifying the overall similarity between proteins, RNAs and their interactions. Combining kernels by tensor product for the set of RNA-protein pairs allowing to share information across the RNA-binding proteins considerably improved the prediction, especially in the case of RNAs with few known binding proteins. Secondly, we have found that contiguous *k*-mer frequencies alone captured rich statistical information on the repetitive conserved motif of RNA-protein pairs and the diversity of amino acids at a sequence has also contributed to an observed improvement contrast to RPI-Pred which just applied 1-letter frequency for both protein and RNA. Finally, a kernel works as a measure of similarity and supports the application of powerful machine learning algorithms such as regularized least squares which we used in this paper. The RLS mehod enables us to efficiently search for an optimized parameter λ at essentially no additional cost [31]. Further, our model was trained on a large data set which contained 2662 RNA-protein pairs, and yielded more robust results. In contrast, IPMiner had much more model parameters to fit as combining deep learning with stacked ensembling, however, was trained on a small data set of just 488 RNA-protein pairs, and thus showed a significantly positive bias on predicting ncRNA-protein interactions. The main disadvantage of the proposed method is that the method is purely data-driven, in the sense that it relies solely on information derived from amino acid sequences and nucleic acid sequences, and thus does not consider higher structural information of protein and RNA. While this may be seen as an advantage, since it can predict any RNA-protein pair of known sequences.

## 4. Materials and Methods

### 4.1. Training Data Set

The increase of the number of protein-RNA complexes in Protein Data Bank [47] has opened possibilities for researchers to develop secondary data bases and to gain valuable insight into the structure and function of these complexes. The Protein-RNA Interface Data base (PRIDB) V2.0 [48] identifies interfacial residues in RNA-protein complexes and also calculates atomic distances between interfacial residues. The RB344 and RB1179 are two precalculated data sets in the PRIDB, which respectively consist of 344 and 1179 RNA-binding protein chains. After combining the RB344 and RB1179 data sets, we obtained a total of 1750 experimentally validated non-redundant RNA-protein pairs, which had at least two atoms respectively coming from RNA and protein with distance no more than 4 Å. Next, we removed redundant RNA-protein pairs, which are the same protein chains interacting with the same RNA chains. Further, we removed those RNA-protein pairs with amino acid sequence length <25 or nucleic acid sequence length <15. Finally, we obtained a positive sample set which consisted of 1331 experimentally validated RNA-protein pairs. So far there are no definite negative samples of RNA–protein interactions that are available. To construct a balanced negative sample set (“RNA-protein non-interacting pairs”), we made it by randomly permute the proteins in the positive sample set but kept the RNA fixed. We repeated the permutation process until no negative pairs existed in the positive sample set. As a result, the training set, called RPI2662, was composed of 1331 RNA-protein interacting pairs and 1331 RNA-protein non-interacting pairs.

### 4.2. Test Data Sets

Several data sets were employed to evaluate the performance of the proposed methods.

Our RPiRLS method was first evaluated using two popular non-redundant data sets of RPIs studied in [27] . The RPI2241 data set consisted of 2241 experimentally validated RNA-protein pairs extracted from the PRIDB data base. While the RPI369 data set eliminated all RPI pairs with ribosomal proteins or ribosomal RNAs from the RPI2241 data set. To avoid overlapping between training and testing data sets, those RPIs overlapping the training data were removed, leaving the RPI2241 data set of 1832 RPIs and the RPI369 data set of 204 RPIs. Their corresponding negative pairs were generated by following the same steps as developing the training negative pairs.

Next, we tested the performance of the RPiRLS method on a large scale data set extracted from the RNA-Protein Interaction Data Base (RPIntDB) (http://pridb.gdcb.iastate.edu/RPISeq/download.php). This data set consisted of 43,010 experimentally validated RPIs from several sources, including the RPIDB, NPInter data base and high-throughput experiments published in literature.

The fourth data set were extracted from the NPInter data base which we called NRPI13153. The NRPI13153 data set consisted of 13,153 experimentally validated ncRNA-protein pairs from six model organisms, i.e., *Caenorhabditis elegans*, *Drosophila melanogaster*, *Escherichia coli*, *Homo sapiens*, *Mus musculus* and *Saccharomyces cerevisiae*. We constructed the fifth data set called LNRPI12114 by extracting only lncRNA-protein pairs from the NPInter data base. This data set contains 12,114 experimentally validated lncRNA-protein pairs.

### 4.3. Methods

**Figure 6:**
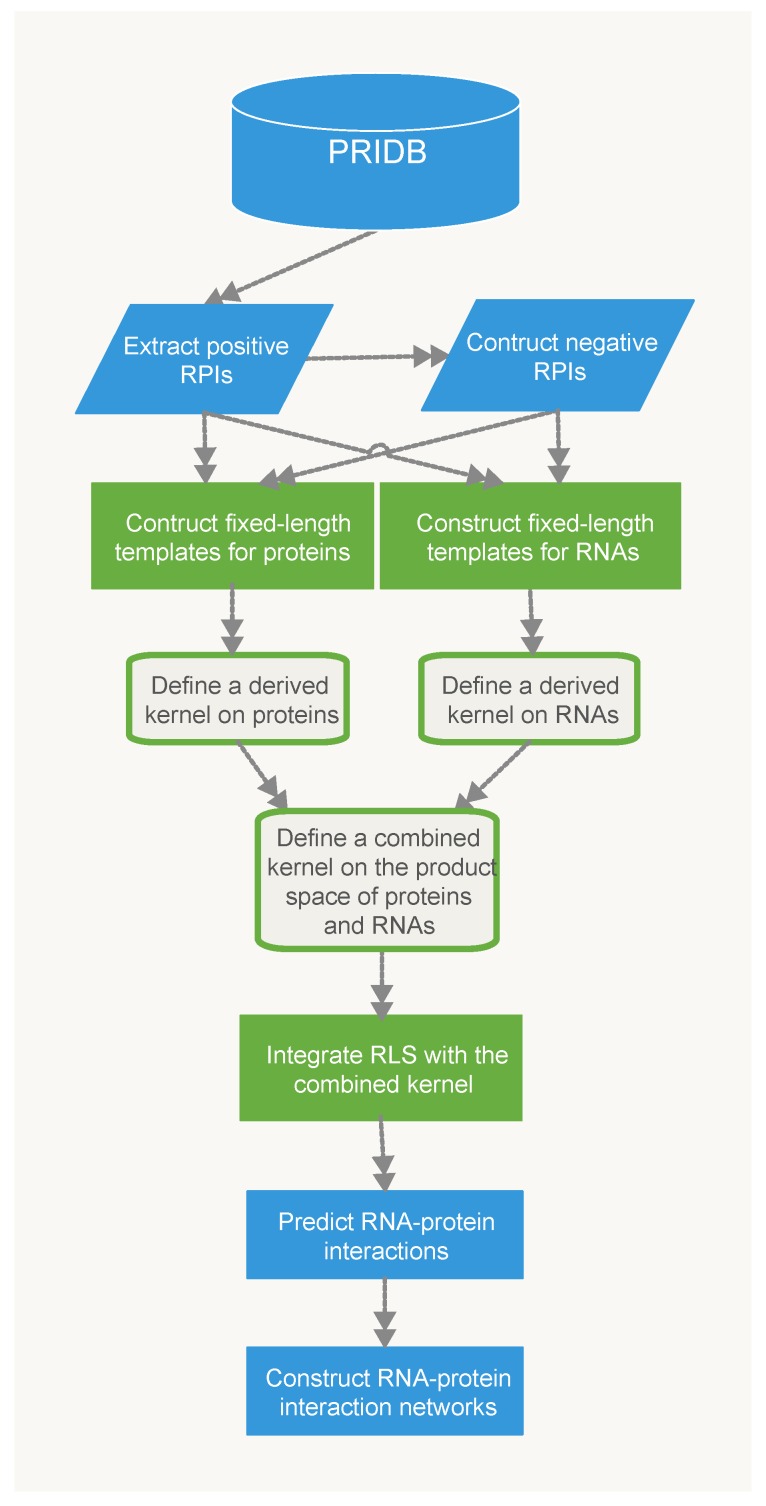
The work flow for the proposed RPiRLS method.

In this paper, we proposed two classifiers for predicting RPIs based on different representations of protein sequences. For the RPiRLS classifier, each amino acid sequence comprised up to 20 different amino acids. While for the RPiRLS-7G classifier, we adopted the same amino acid classification approach as [26,29]. The 20 amino acids were categorized into 7 groups based on their dipole moments and the volume of their side chains: {A, G, V}, {I, L, F, P}, {Y, M, T, S}, {H, N, Q, W}, {R, K}, {D, E}, {C}, and then each amino acid sequence was represented by using the 7-letter reduced alphabets. For both classifiers, they were presented by integrating the 2-layer derived kernel with regularized least squares (RLS) regression algorithm for predicting RNA-protein interaction. The work flow for the development of RPiRLS method was showed in Figure 6.

#### 4.3.1. Derived Kernel

The derived kernel was proposed by Smale et al. [49] on images inspired by neuroscience of visual cortex. In what follows, we briefly described the construction of derived kernel on sequences.

Suppose A is a finite set called the alphabet. In the work here A is the set of 20 amino acids (for RPiRLS), 7 alphabets (for, RPiRLS-7G) or 4 nucleic acids. Let $A^{1}=A$ and define $A^{k+1}=A^{k}\times A$ recursively for any k∈N. We say *s* is a string if $s\in \cup_{k=1}^{\infty }A^{k}$, and $s=(s_{1},\ldots ,s_{k})$ is a *k*-mer (e.g., a sequence of length *k*) if $s\in A^{k}$ for some k∈N with $s_{i}\in A$. The process of computing the derived kernel mainly includes three steps as below:

**Step** **1.** Set an initial kernel ${\hat{K}}_{1}$ at the first layer. Here the initial kernel is defined as:
  (1) $$\begin{array}{c} {\hat{K}}_{1}\left(x,y\right)=\left\{\begin{array}{cc} 1, & x=y\\ 0, & \mathrm{otherwise} \end{array}\right., \end{array}$$
  where $x,y\in A^{k}$. $x=\{x_{1},\ldots ,x_{k}\}$ and $y=\{y_{1},\ldots ,y_{k}\}$ are substrings of the same length *k*. x=y if and only if $x_{i}=y_{i}$ for i=1,…,k.
**Step** **2.** Let $f=(f_{1},\ldots ,f_{n})$, Denote by $\left|f\right|$ the length of *f*, so here $\left|f\right|=n$. Then define the second layer neural response of *f* at *t* :
  (2) $$\begin{array}{c} N_{2}\left(f\right)\left(t\right)=\frac{1}{n-k+1}\sum_{h\in H_{1}}{\hat{K}}_{1}\left(f\circ h,t\right),\;t\in T_{1}, \end{array}$$
  where $T_{1}$ is the template set at the first layer, here we consider all possible substrings of length *k* making up the template set $T_{1}$, so here $T_{1}=A^{k}$. $H_{1}$ is the transformation set at the first layer. $\forall h\in H_{1}$, h:(1,…,k)→(i,…,i+k−1), for 1≤i≤n−k+1. The second layer neural response of *f*, denoted as $N_{2}\left(f\right)$, defines a map as $N_{2}\left(f\right):T_{1}\to \left[0,1\right]$.
**Step** **3.** Compute the second layer derived kernel by normalizing the inner product of two neural responses:
  (3) $$\begin{array}{c} K_{2}\left(f,g\right)={\langle N_{2}\left(f\right),N_{2}\left(g\right)\rangle }_{L^{2}\left(T_{1}\right)}, \end{array}$$
  where ${\langle \cdot ,\cdot \rangle }_{L^{2}\left(T_{1}\right)}$ denotes the $L^{2}$ inner product with respect to the uniform measure $\frac{1}{\left|T_{1}\right|}\sum_{t\in T_{1}}\delta_{t}$, where $\left|T_{1}\right|$ is the cardinality of the template set $T_{1}$ and $\delta_{t}$ is the Dirac measure; $N_{2}\left(f\right)\left(t\right)=\frac{1}{n-k+1}\sum_{h\in H_{1}}{\hat{K}}_{1}\left(f\circ h,t\right),\;t\in T_{1}$.
  With correlation normalization:
  (4) $$\begin{array}{c} {\hat{K}}_{2}\left(f,g\right)=\frac{K_{2}\left(f,g\right)}{\sqrt{K_{2}\left(f,f\right)K_{2}\left(g,g\right)}}. \end{array}$$

This process can continue if appropriate higher level templates are defined. At each layer (local) derived kernels are built by recursively pooling over previously defined local kernels. Here, for the 2-layer derived kernel, pooling is accomplished by taking an average over a set of transformations which calculating the frequency of a template that occurs in a sequence.

In this paper we deal with inner product kernels *K* which satisfies the Mercer Condition, are known to be an instance of reproducing kernels. Next with correlation normalization, $\hat{K}$ is also a reproducing kernel and $\hat{K}\left(x,x\right)=1$ for all x∈X.

The kernel function is symmetric (i.e., K(f,g)=K(g,f)), and non-negative (i.e., $K(f,f^{\prime })\geq 0$), therefore it can be interpreted as a measure of similarity.

We first apply the kernel to the set R which contains nucleic acid sequences, and denote it by ${\hat{K}}_{2}^{R}$, and then apply the kernel to the set of amino acid sequences P, denote it by ${\hat{K}}_{2}^{P}$, and lastly combine two kernels in a natural way by tensor product for the set of RNA-protein pairs . The reproducing kernel for two RNA-protein pairs $\left(r,p\right),\left(r^{\prime },p^{\prime }\right)\in R\times P$ is defined by:

(5) $$\begin{array}{c} {\hat{K}}_{2}^{\mathrm{dk}}\left(\left(r,p\right),\left(r^{\prime },p^{\prime }\right)\right)={\hat{K}}_{2}^{R}\left(r,r^{\prime }\right){\hat{K}}_{2}^{P}\left(p,p^{\prime }\right). \end{array}$$

Since both ${\hat{K}}_{2}^{R}\left(r,r^{\prime }\right)$ and ${\hat{K}}_{2}^{P}\left(p,p^{\prime }\right)$ are positive definite kernels, ${\hat{K}}_{2}^{\mathrm{dk}}\left(\left(r,p\right),\left(r^{\prime },p^{\prime }\right)\right)$ is obviously a positive definite kernel too [50].

After combining the kernel with other kernel-based supervised learning algorithm, we can predict RPIs to any RNA-protein pairs with known primary sequences.

#### 4.3.2. Regularized Least Squares

The RLS algorithm is one of the most widely used models for regression. Let *K* be a kernel on a finite set *X*. Write $H_{K}$ to denote the inner product space of functions on *X* defined by *K*. Suppose $\bar{z}={\left\{\left(x_{i},y_{i}\right)\right\}}_{i=1}^{m}$ is a sample set (called the training set) with $x_{i}\in X$ and $y_{i}\in R$ for each *i*. The RLS can be written as follows:

(6) $$\begin{array}{c} f_{\bar{z},\lambda }=\arg\;\min_{f\in H_{K}}\left\{\frac{1}{\#\bar{z}}\sum_{\left(x_{i},y_{i}\right)\in \bar{z}}{\left(f\left(x_{i}\right)-y_{i}\right)}^{2}+\lambda {∥f∥}_{K}^{2}\right\}. \end{array}$$

#### 4.3.3. Integrate RLS with the Combined Kernel

We integrated RLS with the combined kernel $K={\hat{K}}_{2}^{\mathrm{dk}}$, hence the main construction is to compute

(7) $$\begin{array}{c} \bar{f}=\arg\;\min_{f\in H_{K}}\sum_{i=1}^{m}{\left(f\left(x_{i}\right)-y_{i}\right)}^{2}+\lambda {∥f∥}_{K}^{2}. \end{array}$$

Herein, we aim to develop a novel method to distinguish RNA-protein interaction pairs from non-RNA-protein interaction pairs. Therefore, for the binary classification case with $y_{i}\in \left\{-1,1\right\}$ for each *i*, if $\bar{f}\leq 0$, the predicted class is −1 ( denotes non-interaction), otherwise it is 1 (denotes interaction).

One important step of RLS is to find a “good” value of the regularization parameter λ>0 in Equation (7). They were selected from an optional set Λ by leave-one-out cross-validation [51] on the training data. We never used testing data for parameter selection which is under the risk of over-fitting.

### 4.4. Prediction Measures

The sensitivity (SE) and specificity (SP) are used to measure the ability of identifying positive and negative instances, respectively. They are defined by

$$\mathrm{SE}=\frac{\mathrm{TP}}{\mathrm{TP}+\mathrm{FN}},\;\mathrm{SP}=\frac{\mathrm{TN}}{\mathrm{TN}+\mathrm{FP}},$$

where TP,TN,FP and FN are the number of true positives, true negatives, false positives and false negatives, respectively.

The accuracy which is used to measure the prediction quality, is defined by

$$\mathrm{Accuracy}=\frac{\mathrm{TP}+\mathrm{TN}}{\mathrm{TP}+\mathrm{TN}+\mathrm{FP}+\mathrm{FN}}.$$

The AUC (Area Under the receiver operating characteristic Curve) is further employed to measure the predictive performance, which is 1 for perfect prediction and 0.5 for random prediction.

## Figures and Tables

**Table 1:** Predictive performance of RPiRLS in terms of the AUC on the RPI2662 training set over varying template sizes.

Template Sizes	*l* = 1	*l* = 2	*l* = 3	*l* = 4	*l* = 5	*l* = 6	*l* = 7	*l* = 8
*k* = 1	0.705	0.813	0.850	0.872	0.851	0.832	0.816	0.802
*k* = 2	0.375	0.767	0.853	0.911	**0.926**	0.920	0.915	0.910
*k* = 3	0.219	0.644	0.802	0.881	0.910	0.921	0.924	0.922
*k* = 4	0.202	0.321	0.767	0.854	0.887	0.902	0.912	0.918

The performance of predicting RPIs was evaluated by using 10-fold stratified cross-validation on the RPI2662 data set. Different combinations of parameters *k* and *l* were evaluated. Remark on the symbols of template sizes: *k* stands for template size of amino acid sequences; *l* stands for template size of nucleic acid sequences. The best AUC in the table is marked in bold.

**Table 2:** Predictive performance of RPiRLS in terms of the accuracy on the RPI2662 training set over varying template sizes.

Template Sizes	*l* = 1	*l* = 2	*l* = 3	*l* = 4	*l* = 5	*l* = 6	*l* = 7	*l* = 8
*k* = 1	0.673	0.763	0.812	0.817	0.779	0.769	0.756	0.743
*k* = 2	0.412	0.730	0.796	0.830	**0.830**	0.814	0.800	0.794
*k* = 3	0.261	0.646	0.731	0.784	0.811	0.823	0.821	0.815
*k* = 4	0.243	0.317	0.702	0.747	0.785	0.804	0.816	0.824

Remark on the symbols of template sizes: *k* stands for template size of amino acid sequences; *l* stands for template size of nucleic acid sequences. The best accuracy in the table is marked in bold.

**Table 3:** Predictive performance of, RPiRLS-7G in terms of the accuracy on the, RPI2662 training data set over varying template sizes.

Template Sizes	*l* = 1	*l* = 2	*l* = 3	*l* = 4	*l* = 5	*l* = 6	*l* = 7	*l* = 8
*k* = 1	0.663	0.737	0.776	0.766	0.733	0.656	0.605	0.578
*k* = 2	0.644	0.746	0.792	0.803	0.783	0.770	0.760	0.752
*k* = 3	0.433	0.755	0.796	**0.823**	0.816	0.795	0.791	0.782
*k* = 4	0.347	0.673	0.764	0.805	0.822	0.816	0.803	0.794
*k* = 5	0.262	0.615	0.727	0.779	0.804	0.816	0.821	0.813
*k* = 6	0.242	0.320	0.703	0.754	0.791	0.808	0.815	0.818

The performance of predicting RPIs was evaluated by using 10-fold stratified cross-validation on the RPI2662 data set. Different combinations of parameters *k* and *l* were evaluated. Remark on the symbols of template sizes: *k* stands for template size of amino acid sequences; *l* stands for template size of nucleic acid sequences. The best accuracy in the table is marked in bold.

**Table 4:** Comparision of RPiRLS with other methods on the RPI369 data set in predicting RNA-protein interactions with known structures.

Measurements	RPiRLS	RPiRLS-7G	RPI-Pred	IPMiner
Accuracy	0.85	0.79	0.49	0.50
AUC	0.92	0.90	-	-
Specificity	0.84	0.72	0.34	0.52
Sensitivity	0.86	0.87	0.63	0.48

Remark: ’-’ stands for the AUC score is not available.

**Table 5:** Comparision of RPiRLS with other methods on the RPI2241 data set in predicting RNA-protein interactions with known structures.

Measurements	RPiRLS	RPiRLS-7G	RPI-Pred	IPMiner
Accuracy	0.80	0.67	0.49	0.50
AUC	0.80	0.74	-	-
Specificity	0.82	0.58	0.38	0.20
Sensitivity	0.79	0.76	0.61	0.79

Remark: ’-’ stands for the AUC score is not available.

**Table 6:** Comparing the accuracy of RPiRLS with other methods in predicting non-coding RNA-protein interactions.

Organism	# ncRPIs	RPiRLS	RPiRLS-7G	RPI-Pred
*Caenorhabditis elegans*	36	0.92	0.61	0.92
*Drosophila melanogaster*	95	0.80	0.52	0.88
*Escherichia coli*	202	0.54	0.52	0.90
*Homo sapiens*	8246	0.92	0.74	0.86
*Mus musculus*	3669	0.91	0.80	0.94
*Saccharomyces cerevisiae*	905	0.91	0.83	0.80
Weighted average	13,153	0.91	0.76	0.88

The weighted average accuracy is given by the weighting on the number of RPIs of different organisms over the total.

**Table 7:** Comparing the accuracy of RPiRLS with other methods in predicting long non-coding RNA-protein interactions.

Organism	# lncRPIs	RPiRLS	RPiRLS-7G	RPI-Pred
*Caenorhabditis elegans*	4	1.00	0.75	0.75
*Drosophila melanogaster*	61	0.87	0.69	1.00
*Escherichia coli*	78	0.45	0.45	0.86
*Homo sapiens*	8039	0.93	0.74	0.86
*Mus musculus*	3495	0.92	0.83	0.95
*Saccharomyces cerevisiae*	437	0.94	0.90	0.87
Weighted average	12,114	0.92	0.77	0.89

The weighted average accuracy is given by the weighting on the number of RPIs of different organisms over the total.
